# Structure–Function Relationship Study of a Secretory Amoebic Phosphatase: A Computational-Experimental Approach

**DOI:** 10.3390/ijms22042164

**Published:** 2021-02-22

**Authors:** Celina Terán-Ramírez, Rosa E. Mares-Alejandre, Ana L. Estrada-González, Patricia L. A. Muñoz-Muñoz, Marco A. Ramos-Ibarra

**Affiliations:** Biotechnology and Biosciences Research Group, Faculty of Chemical Sciences and Engineering, Autonomous University of Baja California, Tijuana 22390, Mexico; cteran@uabc.edu.mx (C.T.-R.); ana.laura.estrada.gonzalez@uabc.edu.mx (A.L.E.-G.); lilian.munoz.munoz@uabc.edu.mx (P.L.A.M.-M.)

**Keywords:** HAP/phytase-like phosphatase, recombinant protein production, structure–function characterization, homology-based modeling, *Entamoeba histolytica*

## Abstract

Phosphatases are hydrolytic enzymes that cleave the phosphoester bond of numerous substrates containing phosphorylated residues. The typical classification divides them into acid or alkaline depending on the pH at which they have optimal activity. The histidine phosphatase (HP) superfamily is a large group of functionally diverse enzymes characterized by having an active-site His residue that becomes phosphorylated during catalysis. HP enzymes are relevant biomolecules due to their current and potential application in medicine and biotechnology. *Entamoeba histolytica*, the causative agent of human amoebiasis, contains a gene (*EHI_146950*) that encodes a putative secretory acid phosphatase (*Eh*HAPp49), exhibiting sequence similarity to histidine acid phosphatase (HAP)/phytase enzymes, i.e., branch-2 of HP superfamily. To assess whether it has the potential as a biocatalyst in removing phosphate groups from natural substrates, we studied the *Eh*HAPp49 structural and functional features using a computational-experimental approach. Although the combined outcome of computational analyses confirmed its structural similarity with HP branch-2 proteins, the experimental results showed that the recombinant enzyme (r*Eh*HAPp49) has negligible HAP/phytase activity. Nonetheless, results from supplementary activity evaluations revealed that r*Eh*HAPp49 exhibits Mg^2+^-dependent alkaline pyrophosphatase activity. To our knowledge, this study represents the first computational-experimental characterization of *Eh*HAPp49, which offers further insights into the structure–function relationship and the basis for future research.

## 1. Introduction

Phosphatases are enzymes that cleave the phosphoester bond of various substrates (e.g., proteins, lipids, and sugars) containing phosphorylated residues [1,2,3]. According to the pH at which they have optimal catalysis, the typical classification divides them into acid or alkaline phosphatases [4].

The histidine phosphatase (HP) superfamily (InterPro: IPR029033) is a large group of functionally diverse enzymes sharing a conserved active site that includes a His residue, which becomes phosphorylated during catalysis. This superfamily comprises two branches that share limited sequence similarity. Branch-1 (InterPro: IPR013078) involves a wide variety of enzymes, including fructose-2,6-bisphosphatases and phosphoglycerate mutases. Branch-2 (InterPro: IPR000560) contains mainly acid phosphatases and phytases [5]. HP enzymes are relevant for biomedicine and biotechnology due to their current and potential applications. For instance, the human prostatic acid phosphatase is a biomarker with clinical significance for prostate cancer [6], and phytases have potential as biocatalysts for sustainable agriculture and animal nutrition [7,8,9].

*Entamoeba histolytica* is the protozoan parasite that causes amoebiasis in humans [10]. Its genome encodes a diverse collection of phosphatase enzymes [11]. A thorough in silico analysis revealed 250 non-redundant putative amoebic phosphatases, which were classified into four subclasses: serine/threonine phosphatases (145), tyrosine phosphatases (79), endonuclease/exonuclease/phosphatases (18), and pyrophosphatases (8), based on the standard features found in the catalytic domains (i.e., sequence, structure, phosphoamino acid specificity, and substrate preferences) [12]. Interestingly, within the amoebic tyrosine phosphatases subclass, 19 were identified as putative HP enzymes, including two acid phosphatases (encoded by *EHI_146950* and *EHI_141060*).

Proteomic analysis of isolated amoebic phagosomes showed the active expression of a wide variety of secretory enzymes, including acid phosphatases [13]. Among these, the 49.3-kDa histidine acid phosphatase(HAP)/phytase-like protein encoded by *EHI_146950* (from now on called *Eh*HAPp49) caught our attention due to an apparent lack of information about its precise function. Here, we studied the structure–function relationship of *Eh*HAPp49 using a computational-experimental approach to gain knowledge about its catalytic capabilities and assess its potential as a biocatalyst in sustainable agriculture or animal nutrition, removing phosphates from natural substrates.

## 2. Results and Discussion

### 2.1. E. histolytica Encodes a HAP/Phytase-Like Protein: EhHAPp49

**Figure 1:**
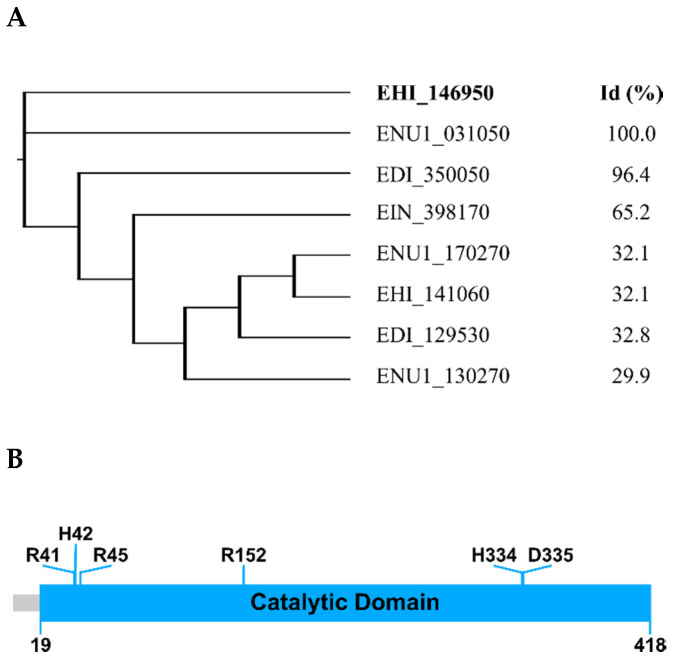
Primary structure of *Eh*HAPp49. (**A**) Dendrogram of Entamoebidae genes encoding protein homologs (**left**) and corresponding pairwise identities (**right**). (**B**) Domain architecture representation. Colored rectangles: signal peptide (**grey**) and phosphatase domain (**blue**). Predicted residues involved in catalysis are also denoted (**top**).

Bioinformatic analysis of the *Eh*HAPp49 primary structure provided the first insights into its structure–function relationship and the initial leads about its enzymatic abilities. As expected, the combined BLAST/CD-Search/InterPro analyses confirmed that it shares significant identities with phosphatases of the HAP/phytase family [5]. Furthermore, a restricted BLAST search of the Entamoebidae database returned several genes encoding homologous proteins (Figure 1A). The polypeptide sequence analysis also revealed that it has a putative N-terminal signal peptide (Met1-Cys18), which suggests protein targeting to the secretory pathway, and a phosphatase domain (Glu19-Gln418), which includes six highly conserved residues (Arg41, His42, Arg45, Arg152, His334, and Asp335) as found in the catalytic core of functional homologs (Figure 1B).

**Figure 2:**
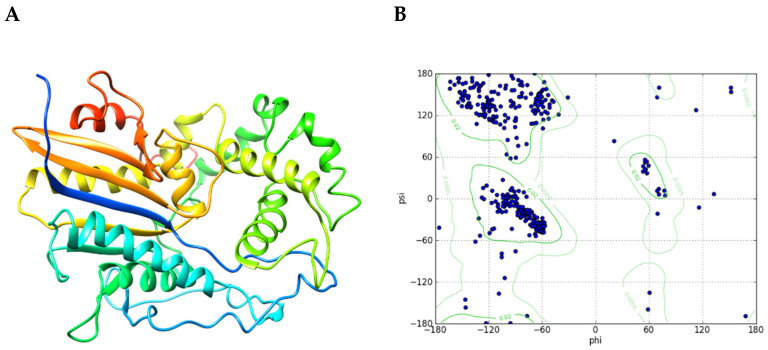
Predicted tertiary structure for *Eh*HAPp49. (**A**) Ribbon representation of the best model. Rainbow-colored from amino (**blue**) to carboxy (**red**). (**B**) Ramachandran plot.

Automatic prediction of the tertiary structure of *Eh*HAPp49 by homology-based modeling offered additional insights into its three-dimensional (3D) conformation. Using the *Eh*HAPp49 polypeptide as the query sequence, a restricted BLAST search of the Protein Data Bank (PDB) repository returned the crystal structures of three protein counterparts as suitable templates (E-value < 10^−14^) for the 3D modeling: (1) the human PAP (prostatic acid phosphatase): 2HPA, 1CVI, 1ND5, and 1ND6 [14,15,16], (2) the human LAP (lysophosphatidic acid phosphatase type 6): 4JOB, 4JOC, and 4JOD [17], and (3) the *Legionella pneumophila* HAP: 5CDH [18]. The top five 3D-models generated by the Modeller multi-template approach met the expected structural benchmarks: low normalized discrete optimized protein energy (zDOPE) score and a Ramachandran plot showing more than 85% of residues in the most favored regions. After refinement using a molecular dynamics (MD)-based method, MolProbity analysis validated the structural accuracy of the best 3D-model for *Eh*HAPp49 (Figure 2A), scoring 2.09 (71st percentile) for protein geometry and 2.79 (98th percentile) for all-atom contacts, which exceeded the benchmark (values >66th percentile are good scores). Moreover, the Ramachandran plot showed that 98.8% of all residues were in the allowed regions, with 93.0% in favored regions (Figure 2B).

Homology-based modeling also predicted disulfide bond formation, a post-translational modification that allows the proper folding and stabilization of numerous secretory proteins [19]. Two disulfide bonds (Cys196-Cys413 and Cys386-Cys394) showing the spatial proximity required for cysteine residue pairing: Cβ-Cβ distance ≤ 4.5 Å [20], are predicted. In addition to stabilization, this structural feature suggests that the native conformation of *Eh*HAPp49 depends on oxidative folding, a cellular process catalyzed by endoplasmic reticulum-resident foldases, such as protein disulfide isomerases [21].

### 2.2. rEhHAPp49 Is an Active Enzyme

Recombinant production of *Eh*HAPp49 and analysis of hydrolytic activity against three common phosphatase substrates provided the first experimental evidence of its enzymatic function. The *EHI_146950*/*Eh*HAPp49 sequence served as a template to design the specific primers used to amplify the fragment encoding the mature polypeptide (Asp14-Gln418). After obtaining the expected amplicon (1239 bp) by high-fidelity PCR, a plasmid engineering approach allowed to clone this product and construct pQEhHAP-Myc22 (Figure 8), which enabled the IPTG-inducible cytosolic expression of r*Eh*HAPp49 as a soluble Myc-tagged protein. Transformed *E. coli* SHuffle cells, harboring pQEhHAP-Myc22, worked as efficient factories for consistent r*Eh*HAPp49 production. Bacterial lysates provided sufficient soluble protein for standard purification by chromatographic procedures (IMAC and gel filtration). The high degree of purity (>95%, as judged by SDS-PAGE analysis) showed the reliability of r*Eh*HAPp49 production. 

A quantitative determination of the enzymatic activity against three reference phosphatase substrates: pNPP, phytic acid, and sodium pyrophosphate (Na-PPi), allowed establishing the catalytic capabilities of r*Eh*HAPp49 under three pH conditions (Table 1). Surprisingly, the enzyme showed negligible HAP/phytase activity at pH 5.0 (acidic conditions), followed by a complete loss at pH 7.0 and 9.0 (neutral and alkaline conditions). In contrast, it exhibited a considerable pyrophosphatase (PPase) activity under acidic conditions, with a significant increase under neutral and alkaline conditions (*p* < 0.001).

### 2.3. rEhHAPp49 Exhibits Pyrophosphatase Activity

**Figure 3:**
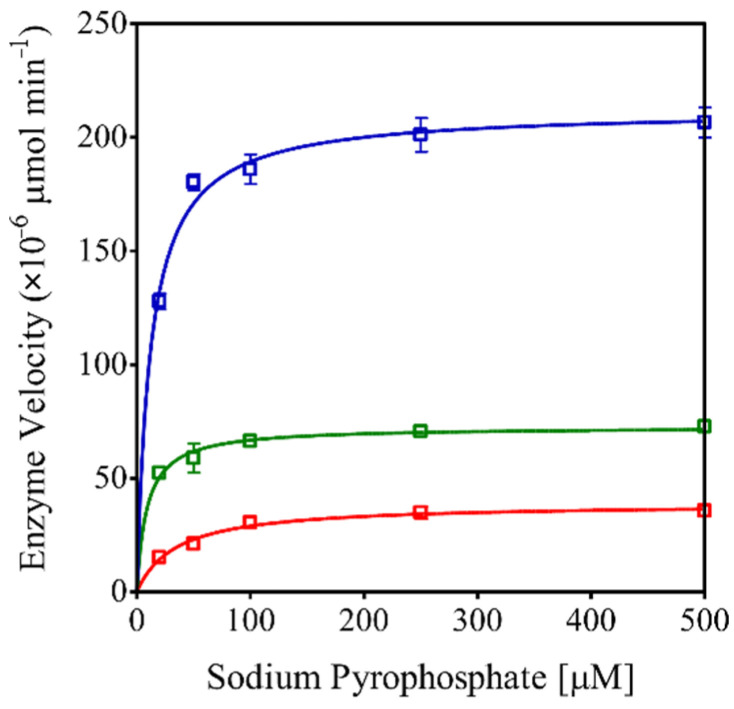
Effect of Na-PPi concentration on the r*Eh*HAPp49 enzyme velocity. PPase activity as a function of pH 5.0 (red), 7.0 (green), and 9.0 (blue). Bars represent the standard error (*n* = 3).

The quantitative determination of PPase activity in r*Eh*HAPp49-assisted reactions with increasing concentrations of Na-PPi (0 to 0.5 mM) under three pH conditions (5.0, 7.0, and 9.0) allowed establishing the effect of substrate concentration on enzyme kinetics. As expected, increasing the Na-PPi concentration boosted the reaction rate, reaching the plateau at 100 µM and exhibiting a maximum velocity at pH 9.0 (Figure 3). Furthermore, the estimated *K_M_* and *k_cat_* values confirmed that r*Eh*HAPp49 shows catalytic efficiency under alkaline conditions (Table 2).

**Figure 4:**
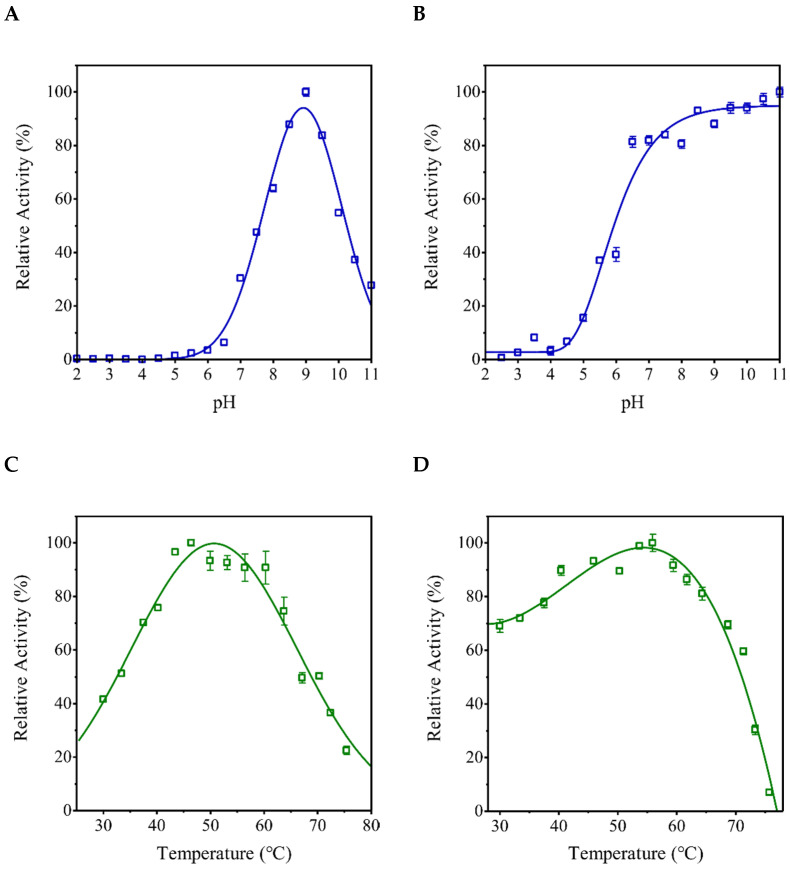
Effects of pH and temperature on activity (**A** and **C**) and stability (**B** and **D**) of r*Eh*HAPp49. Bars represent the standard error (n = 3).

A subsequent evaluation of PPase activity in r*Eh*HAPp49-assisted reactions conducted at different pH (2.0–11.0) or temperature (30–76 °C) conditions allowed establishing the effect of these environmental variables on enzyme activity and stability. As suspected, r*Eh*HAPp49 showed optimal activity at pH 9.0 and ≥50% activity within pH 7.6–10.3 (Figure 4A), implying that the ionization state of the catalytic residues favors enzymatic activity [22]. Furthermore, the high activity (≥90%) retained after 14 h at pH values within the 8.0–11.0 range (Figure 4B) confirmed enzymatic stability under these alkaline conditions. In contrast, the low activity (≤15%) detected under acidic conditions (pH ≤ 5.0) indicated that the protonation of protein residues destabilizes the active site, leading to decreased enzymatic activity [23]. On the other hand, r*Eh*HAPp49 showed optimal activity at 50 °C and suboptimal activity (≥90%) at temperatures within the 44–58 °C range (Figure 4C). Furthermore, the high activity (≥70%) retained after 30 min at temperatures <55 °C confirmed the enzyme thermal-stability (Figure 4D). In contrast, the loss of activity at higher temperatures suggested irreversible heat-induced inactivation.

**Figure 5:**
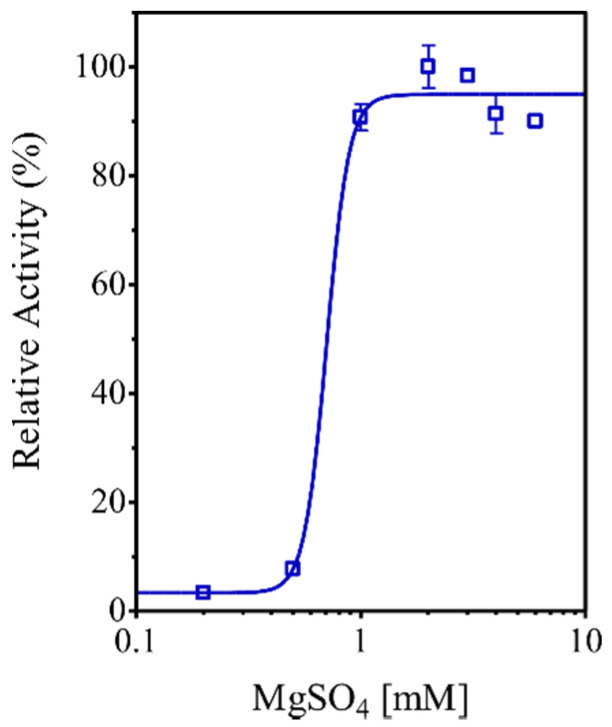
Effect of magnesium ions (Mg^2+^) on the r*Eh*HAPp49 PPase activity. Bars represent the standard error (n = 3).

A supplementary experimental analysis confirmed that the PPase activity showed by the r*Eh*HAPp49 enzyme depends on Mg^2+^ ions as cofactors, with an estimated EC_50_ value for MgSO_4_ of 0.71 ± 0.05 mM (Figure 5).

### 2.4. The Active-Site Entrance of EhHAPp49, an Apparent Molecular Sieve

**Figure 6:**
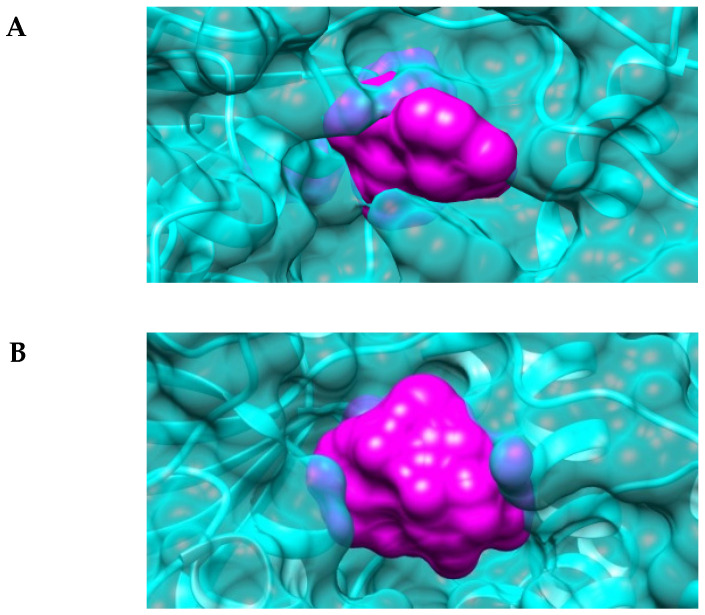
Structural conformation of the predicted active site of *Eh*HAPp49. Surface representation of the substrate-binding pocket of (**A**) *Eh*HAPp49 and (**B**) *Ec*AppA (PDB Entry 1DKP). Colored surfaces: polypeptide chain (cyan) and pocket volume (magenta).

The automatic prediction of the active site conformation followed by a computational analysis provided further insights into the structure–function relationship of *Eh*HAPp49. As expected, the 3D-model showed that it folds similarly to functional homologs. Still, the surface representation offered a better perspective of both the substrate-binding pocket and the active-site entrance. While the pocket retained a typical topography, the entrance shaped a narrow gap (Figure 6A), compared to those of *Ec*AppA (Figure 6B), the bacterial phytase [24,25]. This observation suggested that a molecular sieving process could be involved in the substrate selectivity shown by r*Eh*HAPp49.

A supplementary analysis by molecular docking simulations supported the latter remark. As suspected, the *Eh*HAPp49 active site entrance acted as a selective sieve, allowing the passage and stable docking of PPi (−6.82 kcal/mol, binding energy) but not doing so for the other ligands (pNPP and phytic acid). Given this theoretical finding, experimental studies will be crucial to accurately test the suggested hypothesis (i.e., the active site entrance plays a structural role in *Eh*HAPp49 substrate-selectivity); for instance, site-directed mutagenesis followed by the analysis of enzyme function [26].

### 2.5. EhHAPp49 Is a Non-Canonical Phosphatase

So far, our findings suggest an apparent discrepancy between the observed and expected function for *Eh*HAPp49. In brief, the enzymatic characterization showed that it lacks the HAP/phytase activity predicted by the bioinformatic analysis; instead, it exhibits a PPase-like activity. Due to this feature and the absence of previous reports showing a similar catalytic transition for any other amoebic enzyme involved in phosphate metabolism, we propose that *Eh*HAPp49 represents a non-canonical phosphatase.

Given the Mg^2+^-dependent alkaline PPase activity shown by r*Eh*HAPp49, a comparative analysis against typical PPases offered the first insight into such protein function. Through enzyme-catalyzed hydrolysis of PPi, cells gain a thermodynamic boost for numerous synthetic reactions [27,28,29]. Typically, PPases (EC 3.6.1.1) are classified as either membrane-bound (M-PPases) or soluble (S-PPases), with an additional subclassification of S-PPases into three families: I, II, and III [28,30]. In terms of catalysis, M-PPases exhibit relatively low rates (≈10 s^−1^), while the S-PPases show three different rates: ≈0.16–0.32 s^−1^ for family III (the lowest), ≈200 s^−1^ for the family I (moderate), and ≈2000 s^−1^ for family II (the highest) [28,31,32,33]. As a relative criterion, the determined rate for *Eh*HAPp49 (≈0.18 s^−1^) suggested a catalytic performance like that defined for S-PPases belonging to family III, i.e., haloacid dehalogenase (HAD) PPases. However, because *Eh*HAPp49 lacks the structural and catalytic motifs commonly shared by HAD PPases [34,35], we consider that it exhibits a non-typical PPase activity.

**Figure 7:**
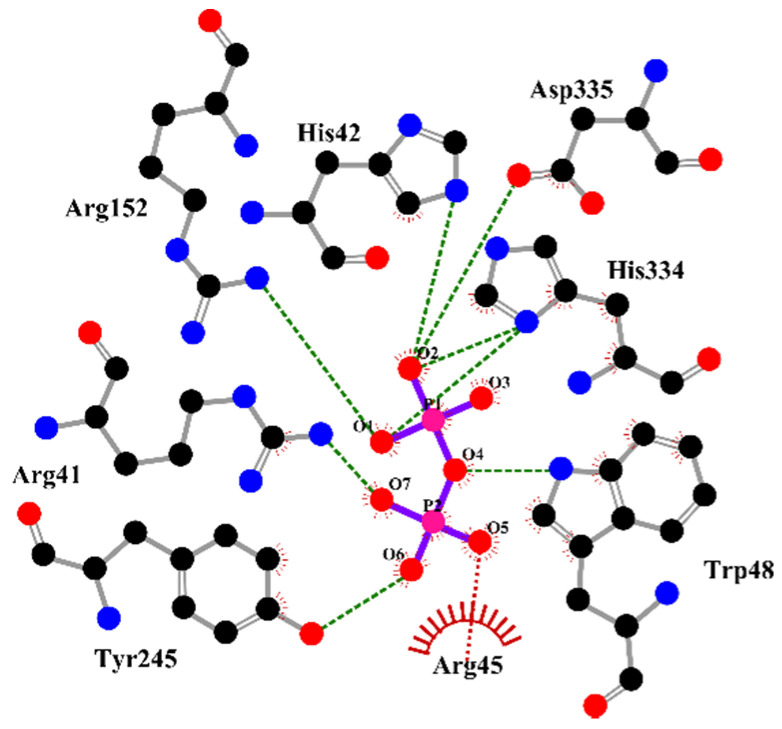
Schematic representation (2D) of residues in the substrate-binding site of *Eh*HAPp49 exhibiting interactions with inorganic pyrophosphate. Color codes: hydrogen bonds, green dashes; salt bridges, red dashes/arcs; carbon, black; oxygen, red; nitrogen, blue; phosphorous, pink; ligand bonds, purple; protein bonds, gray.

Furthermore, supplementary analysis of the substrate–enzyme interactions provided additional insights into the *Eh*HAPp49 active-site structure. S-PPases usually require 3–4 metal ions for maximal activity (depending on the pH and enzyme family), while most residues in the active site have supportive roles (as reactions catalyzed by these proceed without an enzyme-phosphate intermediate) [28,36,37]. In addition to the already proved dependence on magnesium ions, its hypothetical ability to bind PPi through numerous non-covalent interactions with active site residues, predicted by molecular docking simulations: Arg41, His42, Arg45, Trp48, Arg152, Tyr245, His334, and Asp335 (Figure 7), suggest that the *Eh*HAPp49 active-site structure contains the molecular requirements to support PPi binding and achieve consistent PPase activity. However, it remains uncertain whether the associated catalytic reaction involves forming an enzyme-phosphate intermediate, as occurs in typical HP enzymes but not in S-PPases.

As a final thought, and based on its biochemical nature (i.e., secretory protein), we reasonably suggest that *Eh*HAP49 could be involved in specialized extracellular processes required for membrane interactions associated with the *E. histolytica* phagocytic activity. Previous studies on the protein structure and function of a pseudophosphatase, called Cf60, secreted by the slime mold *Dictyostelium discoideum* support this hypothesis. Remarkably, Cf60 has structural similarity to HAP/phytase enzymes (branch-2 of the HP superfamily), but it lacks acid phosphatase activity. Nonetheless, as a component of the counting factor (CF, a 450-kDa protein complex secreted by *D. discoideum* cells), it regulates the multicellular-structure size. Furthermore, functional analysis based on gene disruption suggested that Cf60 is essential for early development [38,39]. Given these, cellular studies will be essential to test the suggested hypothesis and determine the functional role of *Eh*HAPp49 in the pathobiology of *E. histolytica*. Likewise, experimental approaches aimed at gaining insights into the structure and function of bioinformatically-identified *Eh*HAPp49 protein homologs will be critical to support the hypothesis of a novel class of phosphatases, likely specific to the phylum Amoebozoa.

## 3. Materials and Methods

### 3.1. Database Searching and Protein Structure Analyses

The *Eh*HAPp49 gene and protein data were retrieved from two servers: AmoebaDB (https://amoebadb.org/amoeba/, accessed on 30 September 2020) and UniProtKB (https://www.uniprot.org/, accessed on 30 September 2020). The NCBI’s BLAST engine (basic local alignment search tool, https://blast.ncbi.nlm.nih.gov/, accessed on 30 September 2020) [40] and CD-Search web tool (https://www.ncbi.nlm.nih.gov/Structure/, accessed on 30 September 2020) [41], as well as the EBI’s InterPro search engine (https://www.ebi.ac.uk/interpro/, accessed on 30 September 2020) [42], were used for the primary structure and conserved domain analyses. Modeller [43], run in UCSF Chimera software [44], was used for the multiple-template homology-based modeling. A restricted BLAST protein search of the PDB repository and Chimera’s Multalign Viewer tool were applied to identify the structural templates and generate the multi-sequence alignment (Supplementary Figure S1). The locPREFMD tool [45], a molecular dynamics (MD)-based computational method for protein structure refinement, was used to improve the local stereochemistry of the best-ranked model (http://feig.bch.msu.edu, accessed on 15 October 2020). The MolProbity web service [46], a bioinformatic tool for all-atom structure validation (http://molprobity.biochem.duke.edu/, accessed on 20 October 2020), was used to verify the 3D-model accuracy.

### 3.2. Materials

Reagents for bacteriological culture media were obtained from Becton, Dickinson and Company (Franklin Lakes, NJ, USA). PCR amplification biochemicals, kits for DNA isolation, and materials for 6xHis-tagged protein purification were obtained from Qiagen (Germantown, MD, USA). Enzymes for standard cloning were obtained from New England Biolabs (Ipswich, MA, USA). Chemicals for protein analysis by SDS-PAGE and immunoblotting were obtained from Bio-Rad Laboratories (Hercules, CA, USA). Unless otherwise specified, all other materials were obtained from Sigma-Aldrich (St. Louis, MO, USA) or GE Healthcare Bio-Sciences (Pittsburgh, PA, USA).

### 3.3. Strains, Plasmids and Primers

Table 3 shows the *Escherichia coli* strains, bacterial plasmids, and synthetic primers used throughout this study. *E. coli* ER2738 was the host strain for plasmid DNA propagation, and *E. coli* SHuffle was the expression strain for recombinant protein production (i.e., r*Eh*HAPp49). Bacterial cells were cultured in Luria-Bertani (LB) broth at 37 °C, with constant shaking (300 rpm). Selection of stably transformed bacteria was performed by antibiotic resistance, using ampicillin (150 µg/mL) or chloramphenicol (15 µg/mL) as required. The pBluescript SK(−) and pBAD33 plasmids were used for standard molecular cloning [47], while pQE30 was the expression plasmid for the recombinant protein production. Synthetic oligonucleotides (Eurofins Genomics LLC, Louisville, KY, USA) were used as primers for PCR amplification.

### 3.4. EhHAPp49 PCR Amplification

The *E. histolytica* HM1:IMSS strain was grown at ~90% confluence in TYI-S-33 medium [48]. The gDNA of 2.5 × 10^6^ amoebic cells was purified using a QIAmp^®^ DNA Mini Kit (Qiagen). An Expand™ High Fidelity PCR System (Sigma-Aldrich) was used to amplify the amoebic gene fragment coding for the *Eh*HAPp49 mature polypeptide (UniProt C4M8S6: Asp14-Gln418), with EHHAP[F/R] as the gene-specific primer set. The PCR cycling conditions were an initial denaturation step (2 min at 94 °C) followed by 10 cycles of exponential amplification involving 20 s at 94 °C, 20 s at 50 °C, and 90 s at 72 °C, 25 cycles of exponential amplification involving 20 s at 94 °C, 20 s at 50 °C, and 110 s at 72 °C, and a final elongation step (7 min at 72 °C). Agarose gel electrophoresis was applied to analyze the amplicon (1239 bp). After that, a QIAquick^®^ PCR Purification Kit (Qiagen) was used to purify the PCR product (i.e., *Eh*HAPp49).

### 3.5. Construction of Recombinant Plasmids Harboring EhHAPp49

BamHI/XhoI sticky-end cloning of *Eh*HAPp49 into pBPelB-BHX-Myc (Supplementary Figure S2) was the approach used to construct pBPelB-EhHAP-Myc (Supplementary Figure S3a). Following proper digestion of both insert and vector (and successive purification of restriction products), sticky-end ligation was catalyzed by T4 DNA ligase (New England Biolabs) in a typical reaction mix. After heat shock-induced transformation of chemically competent bacteria and subsequent selection of transformants, performed following standard protocols, the recombinant plasmid was isolated by DNA purification using a QIAprep^®^ Spin Miniprep Kit (Qiagen).

XbaI/HindIII sticky-end cloning of PelB-*Eh*HAPp49-Myc into pBAD33 [49] was the consecutive approach to construct pBAD-PelB-EhHAP-Myc (Supplementary Figure S3b). With M13_RV/H3MYCR as the primer pair and pBPelB-EhHAP-Myc as a template, the insert (1475 bp) was amplified by high-fidelity PCR, using both reaction system and cycling conditions as defined previously. After proper digestion-purification of both insert and vector, the subsequent molecular protocols (i.e., ligation of sticky ends, selection of transformed bacteria, and purification of plasmid DNA) were performed by standard methods, as mentioned above.

Each construct was selected by endonucleolytic analysis, followed by verification of the cloned fragment through DNA sequencing.

### 3.6. Construction of the Recombinant Plasmid Expressing EhHAPp49

**Figure 8:**
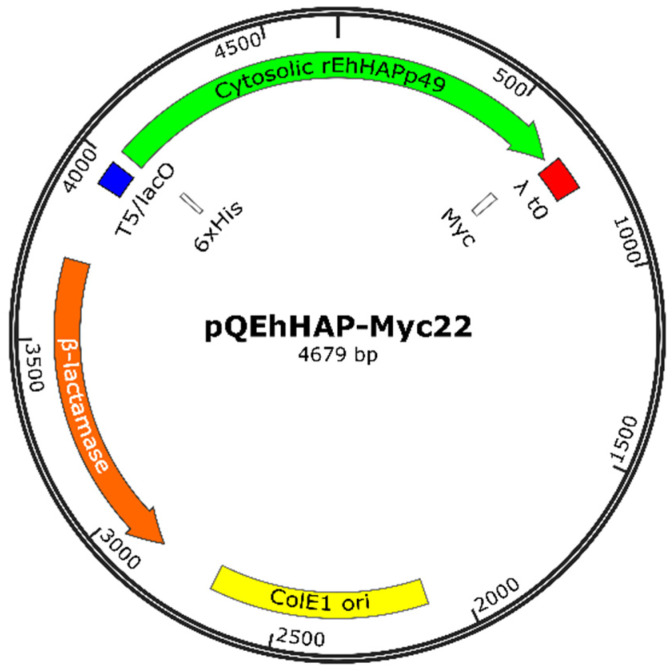
Schematic representation of the pQEhHAP-Myc22 plasmid, which encodes *Eh*HAPp49 as a 6xHis/Myc-tagged cytosolic protein. ColE1 ori (yellow) functions as the autonomous replication sequence, and the encoded β-lactamase (orange) as a selection marker (Amp^R^). As a transcriptional unit, the promoter and terminator: T5/lacO (blue) and λ t0 (red), regulate the gene expression of r*Eh*HAPp49 through an IPTG-inducible system.

BamHI/HindIII sticky-end cloning of *Eh*HAPp49-Myc into pQE30 (Qiagen), a bacterial vector that allows high-level expression of His-tagged recombinant proteins, was the approach used for constructing pQEhHAP-Myc22 (Figure 8). The Expand™ High Fidelity PCR System was used to amplify the insert (1630 pb), with pBAD-PelB-EhHAP-Myc as the template and BAD_[FW/RV] as the primer pair. Molecular cloning, from insert/vector digestion to DNA sequencing, was conducted by standard protocols.

### 3.7. Production of rEhHAPp49

*E. coli* SHuffle cells harboring pQEhHAP-Myc22 were the microbial factories used to produce r*Eh*HAPp49 (i.e., 6xHis-*Eh*HAPp49-Myc). A 2-mL overnight culture sample was the inoculum for each production culture batch (100 mL). The standard procedure for r*Eh*HAPp49 production involved bacteria culturing at 37 °C for a 2-h period, with shaking (300 rpm), followed by adding isopropyl β-D-1-thiogalactopyranoside (IPTG, 0.5 mM final concentration) and further culturing at 30 °C for 16 h (300 rpm). Subsequently, a centrifugation run (9300× *g*; 10 min; 10 °C) was applied to obtain proper cell pellets for r*Eh*HAPp49 purification.

CelLytic^®^ B reagent (Sigma-Aldrich), supplemented as recommended by the supplier (100 U/mL benzonase, 0.2 mg/mL lysozyme, and 1X protease inhibitor cocktail), was used to lyse the bacterial cells. Next to suspension in this reagent (5 mL), cells were disrupted by sonication (10 cycles: 30 s ON, 30 s OFF) in an ice-bath. The protein extraction process was completed by slow shaking for 10 min. Two consecutive centrifugations isolated the soluble fraction: a mid-speed run (9300× *g*; 15 min; 10 °C) to remove the cell debris and a high-speed run (16,000× *g*; 15 min; 10 °C) to separate the fine sediment.

The expression product (r*Eh*HAPp49) was purified through immobilized-metal affinity chromatography (IMAC) and desalted using a Sephadex G-25 column. Ni-NTA agarose (Qiagen) was the matrix used for IMAC (1-mL bed volume). The soluble fraction was first diluted with a volume of buffer D (600 mM NaCl; 40 mM imidazole-HCl, pH 8.0; 40 mM Tris-HCl, pH 8.0) and then loaded onto the column. After extensive washing with buffer A (300 mM NaCl; 20 mM imidazole-HCl, pH 8.0; 20 mM Tris-HCl, pH 8.0), 5 bed volumes of buffer B (300 mM NaCl; 250 mM imidazole-HCl, pH 8.0; 20 mM Tris-HCl, pH 8.0) were used to elute the His-tagged recombinant protein. A concentrate of r*Eh*HAPp49 was obtained by pooling those chromatographic fractions with large amounts of pure protein. Next, a PD-10 column (GE Healthcare Bio-Sciences) was used for desalting as recommended by the manufacturer. The elution/preservation buffer for pure r*Eh*HAPp49 was 20 mM Tris-HCl (pH 8.0).

Production of r*Eh*HAPp49 was monitored by routinely performing a typical SDS-PAGE [50] analysis (Supplementary Figure S4), and protein concentration was determined using the Bradford colorimetric assay [51].

### 3.8. EhHAPp49 Enzyme Activity Assays

The enzymatic function of r*Eh*HAPp49 was studied using three phosphatase substrates (as reference compounds): p-nitrophenyl phosphate (pNPP), phytic acid, and sodium pyrophosphate (Na-PPi). Standard activity assays were performed either under acidic, neutral, and alkaline conditions. A 100 mM solution of the respective buffer: Na-acetate (pH 5.0; acid) or Tris-HCl (pH 7.0/9.0; neutral/alkaline), was used to set each condition. The hydrolytic reactions were allowed for 60/180 mins at 37 °C. Reaction mixes (200 µL final) and specific settings for each activity assay (i.e., phosphatase, phytase, and pyrophosphatase) were as follows.

#### 3.8.1. Phosphatase Activity Assay

The enzyme (1 µM r*Eh*HAPp49) and cofactor (5 mM MgSO_4_) were incubated for 15 min at 37 °C before adding the substrate (10 mM pNPP). After a 60-min period of enzyme-assisted hydrolysis, the reaction was stopped by rapidly mixing with 100 µL of 1.2 N NaOH. After that, the supernatant was isolated through centrifugation at 16,000× *g* for 1 min. An enzyme-free reaction (i.e., uncatalyzed) was used as a blank to subtract the background. The absorbance (A_415_) was promptly measured and used to determine the p-nitrophenolate (pNP) concentration with a standard curve. The amount of pNP (µmole) released per minute per µmole of r*Eh*HAPp49 defined the specific phosphatase activity.

#### 3.8.2. Phytase Activity Assay

Before adding the substate (1.5 mM phytic acid), the enzyme/cofactor mix was prepared and treated as described above. After 180 min of enzyme-assisted hydrolysis, the reaction was immediately cooled in an ice-bath and stooped by thoroughly mixing with 100 µL of 6% trichloroacetic acid. The supernatant was then separated by centrifugation at 16,000× *g* for 15 min (10 °C). A Pi-background reaction, in which the substrate was added immediately after the stopping solution, functioned as a blank. The malachite green (MG) colorimetric assay [52], detailed below, was used to determine the Pi concentration. The amount of Pi (µmole) released per minute per µmole of r*Eh*HAPp49 defined the specific phytase activity.

#### 3.8.3. Pyrophosphatase Activity Assay

Before adding the substrate (0.5 mM Na-PPi), the enzyme/cofactor mix was prepared and treated as previously stated. After a 60-min period of enzyme-assisted hydrolysis, the subsequent steps (from reaction stopping to Pi determination) were as detailed in the phytase assay. The amount of Pi (µmole) released per minute per µmole of r*Eh*HAPp49 defined the specific PPase activity.

#### 3.8.4. MG Colorimetric Assay

*MG colorimetric assay*. MG color reagent batch was freshly-prepared based on the assay requirements by mixing the following components: 20 parts of 0.13% MG (in 3.1 M H_2_SO_4_), 5 parts of 7.8% ammonium molybdate, and 1 part of 5.2% Tween-20. After 30 min standing at room temperature, the mix was then centrifuged at 16,000× g for 10 min to remove the fine sediment. For the assay, 25 µL of MG reagent and 100 µL of supernatant (from the phytase or PPase activity assay) were thoroughly mixed, and color development was allowed for 10 min (exact) at room temperature. Absorbance (A_650_) was immediately measured and used to determine the Pi concentration with a standard curve.

### 3.9. Characterization of the EhHAPp49 PPase Activity

The effect of substrate on enzyme kinetics was assessed by conducting the PPase assay at different concentrations of Na-PPi (0–0.5 mM) under acidic, neutral, and alkaline conditions (established as previously mentioned). Reaction mixes (200 µL final) included 0.1 µM r*Eh*HAPp49 and 5 mM MgSO_4_. Kinetics parameters, *K_M_* and *k_cat_*, were determined by fitting the data (i.e., enzyme velocity against substrate concentration) to a nonlinear least-squares regression model using the Michaelis–Menten equation.

The effect of pH on enzyme activity was evaluated by performing the PPase assay as previously stated but using distinct buffers to establish the pH conditions: Gly-HCl (pH 2.0–3.5), Na-acetate (pH 4.0–5.0), MES-NaOH (pH 5.5–6.5), Tris-HCl (pH 7.0–9.0), and Gly-NaOH (pH 9.5–11.0). The optimum pH for activity was established by fitting the data (i.e., PPase against pH) to a dose–response curve using a bell-shaped Gaussian distribution model. The effect of pH on enzyme stability was assessed by incubating r*Eh*HAPp49 under different pH conditions: 2.0–11.0, for 14 h (4 °C) before assaying the PPase activity. The pH thresholds for stability were determined by fitting the data (i.e., PPase against pH) to a dose–response curve using a sigmoidal distribution model.

The effect of temperature on enzyme activity was assessed by performing the PPase assay in the 30–76 °C range using the reaction settings described above, but under alkaline conditions (pH 9.0). The optimum temperature for activity was determined by fitting the data (i.e., PPase against temperature) to a dose–response curve using a bell-shaped Gaussian distribution model. The effect of temperature on enzyme stability was assessed by preincubating r*Eh*HAPp49 under distinct thermal conditions: 30–76 °C for 30 min (pH 9.0), before assaying PPase activity. The temperature thresholds for stability were determined using the method described earlier. A MultiGene™ Thermal Cycler (Labnet International, Inc.; Edison, NJ, USA) was used to control the temperature.

The effect of magnesium ions on PPase activity was evaluated by conducting the enzymatic reaction under optimal conditions with increasing concentrations of MgSO_4_ (0–5 mM). The EC_50_ value was established by fitting the data (i.e., PPase against the log10 value of MgSO_4_ concentration) to a dose–response curve using a sigmoidal distribution model.

### 3.10. Data Analysis

All data represent the mean (±standard error) of three independent experiments. One-way analysis of variance (ANOVA) with a post-hoc Tukey test was used for multiple comparisons. GraphPad Prism^®^ 4.0 for Windows (San Diego, CA, USA) was the computational package used for all statistical analyses.

### 3.11. In Silico Analysis of the EhHAPp49 Ligand-Binding Site

IntFOLD (https://www.reading.ac.uk/bioinf/IntFOLD/, accessed on 1 October 2020), an integrated interface for protein structure and function prediction [53,54], was used to model the *Eh*HAPp49 active site and estimate the presumed protein-ligand interactions. While FunFOLD was used to predict the ligand-binding site residues, using ligand-containing structures (from the PDB database) as templates and the default settings for homology-based modeling [55,56], FunFOLDQA was used to assess the 3D-model accuracy [57]. The top-ranked model of the *Eh*HAPp49 active site (saved as a PDB file) was used as the receptor for molecular docking simulations with ArgusLab (Planaria Software LLC; Seattle, WA) [58]. Ligands (pNPP, phytic acid, and PPi) were simulated based on 3D structures (SDF files), retrieved from PDB (https://www.rcsb.org/search/advanced, accessed on 5 October 2020) and converted to Mol files with OpenBabel 3.0 [59]. ArgusDock was used as the shape-based algorithm for flexible ligand docking with default settings for geometry optimizations and energy calculations.

PyMOL (Schrödinger, LLC; New York, NY, USA) and UCSF Chimera were the interactive molecular graphics systems used for structural analysis, whereas LigPlot+ [60] and PLIP [61] were visualization tools used to analyze the protein ligand-binding site.

## 4. Conclusions

Here, we studied the structural and biochemical features of the amoebic enzyme *Eh*HAPp49 using a computational-experimental approach. Bioinformatic analyses of genomic and proteomic data confirmed its similarity to HAP/phytases (branch-2 of the HP superfamily). We engineered a bacterial plasmid for cytosolic expression of the recombinant enzyme (r*Eh*HAPp49) after induction with IPTG. A standard protocol for protein production in *E. coli* cells yielded a suitable amount of soluble-active r*Eh*HAPp49 for the biochemical characterization. Based on the enzymatic characterization and supported by supplementary in silico studies, we determined that *Eh*HAPp49 is a non-canonical phosphatase that exhibits non-typical PPase activity. Overall, our findings provide additional knowledge about the structure and function of *Eh*HAPp49 and offer the basis for future research. For instance, the preference for other organic or linear inorganic polyphosphate substrates, such as thiamine pyrophosphate, adenosine (di/tri)phosphate, or tripolyphosphate, should be assessed to establish a precise protein function (i.e., enzyme specificity). Furthermore, as a closing thought, it is reasonable to assume that *Eh*HAPp49 could belong to a novel class of phosphatases, likely specific to the phylum Amoebozoa.

## Figures and Tables

**Table 1:** Phosphatase, phytase, and pyrophosphatase activities of r*Eh*HAPp49.

pH (Buffer ^§^)	Substrate		
	p-Nitrophenyl Phosphate ^1^	Phytic Acid ^2^	Sodium Pyrophosphate ^3^
5.0 (Na-acetate)	0.05 ± 0.005	0.02 ± 0.002	0.2 ± 0.02
7.0 (Tris-HCl)	Not significant	Not significant	2.8 ± 0.04
9.0 (Tris-HCl)	Not significant	Not significant	9.3 ± 0.05

^§^ 100 mM (final). ^1^ Specific phosphatase activity: the amount of p-nitrophenolate (µmole) released per min per µmole of r*Eh*HAPp49. ^2^ Specific phytase activity: the amount of inorganic phosphate (µmole) released per min per µmole of r*Eh*HAPp49. ^3^ Specific PPase activity: the amount of inorganic phosphate (µmole) released per minute per µmole of r*Eh*HAPp49.

**Table 2:** Michaelis–Menten kinetic parameters of the pyrophosphatase activity of r*Eh*HAPp49.

pH	*K_M_* (µM)	*k_cat_* (min^−1^)	*k_cat_*/*K_M_* (µM^−1^ min^−1^) ^§^
5.0	34 ± 6	0.2 ± 0.0	0.006
7.0	9 ± 2	3.6 ± 0.1	0.400
9.0	12 ± 2	10.6 ± 0.2	0.883

^§^ Ratio of mean values.

**Table 3:** Strains, plasmids, and primers used in this study.

Strain	Genotype	Source
ER2738	*F´ proA^+^B^+^ lacIq Δ(lacZ)M15 zzf::Tn10(TetR) / fhuA2 glnV Δ(lac-proAB) thi-1 Δ(hsdS-mcrB)5*	NEB ^1^
SHuffle	*fhuA2 [lon] ompT ahpC gal λatt::pNEB3-r1-cDsbC (Spec^R^, lacI^q^) ΔtrxB sulA11 R(mcr-73::miniTn10-Tet^S^)2 [dcm] R(zgb-210::Tn10 -Tet^S^) endA1 Δgor ∆(mcrC-mrr)114::IS10*	NEB ^1^
**Plasmid**	**Features**	**Source**
pBluescript SK(-)	Lactose regulation, ColE1 origin, Amp^R^	Stratagene
pBPelB-BHX-Myc	pBluescript-based, PelB signal, Myc tag	This study
pBPelB-EhHAP-Myc	pBluescript-based, periplasmic *Eh*HAPp49 (Myc-tagged)	This study
pBAD33	Arabinose regulation, p15A origin, Cm^R^	ATCC ^2^
pBAD-PelB-EhHAP-Myc	pBAD33-based, periplasmic *Eh*HAPp49 (Myc-tagged)	This study
pQE30	Lactose regulation, ColE1 origin, Amp^R^	Qiagen
pQEhHAP-Myc22	pQE30-based, cytosolic *Eh*HAPp49 (6xHis- and Myc-tagged)	This study
**Primer**	**Sequence (5´ to 3´)**	**Endonuclease**
EHHAPF	catcat*ggatcc*gatttaacatactgtgaagtacctgaa tt	*BamHI*
EHHAPR	catcat*ctcgag*ctgatttttggcattacagtctga	*XbaI*
M13_RV	caggaaacagctatgac	None
H3MYCR	catcat*aagctt*ttacagatcctcttcagagatgagt	*HindIII*
BAD_FW	cggcgtcacactttgctatgc	None
BAD_RV	tgggaccaccgcgctactgcc	None

^1^ New England Biolabs (Ipswich, MA, USA), ^2^ American Type Culture Collection (Manassas, VA, USA).

## Data Availability

The data presented in this study are available on request from the corresponding author, without undue reservation, to any qualified researcher.

## Supplementary Materials

The following are available online at https://www.mdpi.com/1422-0067/22/4/2164/s1, Figure S1. Multiple sequence alignment of the protein sequences of EhHAPp49 and three structural homologs, Figure S2: Schematic depiction of pBPelB-BHX-Myc, Figure S3: Schematic depictions of pBPelB-EhHAP-Myc and pBAD-PelB-EhHAP-Myc, Figure S4: Analysis of IMAC fractions by 13.5% SDS-PAGE.

## References

[1] Dick C.F., Dos-Santos A.L.A., Meyer-Fernandes J.R. (2011). Inorganic Phosphate as an Important Regulator of Phosphatases. Enzyme Res..

[2] Fontanillo M., Köhn M. (2016). Phosphatases: Their Roles in Cancer and Their Chemical Modulators. Adv. Exp. Med. Biol..

[3] Dotaniya M.L., Aparna K., Dotaniya C.K., Singh M., Regar K.L. (2019). Role of Soil Enzymes in Sustainable Crop Production. Enzymes in Food Biotechnology.

[4] Vincent J.B., Crowder M.W., Averill B.A. (1992). Hydrolysis of Phosphate Monoesters: A Biological Problem with Multiple Chemical Solutions. Trends Biochem. Sci..

[5] Rigden D.J. (2008). The Histidine Phosphatase Superfamily: Structure and Function. Biochem. J..

[6] Xu H., Wang F., Li H., Ji J., Cao Z., Lyu J., Shi X., Zhu Y., Zhang C., Guo F. (2019). Prostatic Acid Phosphatase (PAP) Predicts Prostate Cancer Progress in a Population-Based Study: The Renewal of PAP?. Dis. Markers.

[7] Bhavsar K., Khire J.M. (2014). Current Research and Future Perspectives of Phytase Bioprocessing. RSC Adv..

[8] Reddy C.S., Kim S.-C., Kaul T. (2017). Genetically Modified Phytase Crops Role in Sustainable Plant and Animal Nutrition and Ecological Development: A Review. 3 Biotech.

[9] Jatuwong K., Suwannarach N., Kumla J., Penkhrue W., Kakumyan P., Lumyong S. (2020). Bioprocess for Production, Characteristics, and Biotechnological Applications of Fungal Phytases. Front. Microbiol..

[10] Kumanan T., Sujanitha V., Sreeharan N. (2020). Amoebic Liver Abscess: A Neglected Tropical Disease. Lancet Infect. Dis..

[11] Loftus B., Anderson I., Davies R., Alsmark U.C.M., Samuelson J., Amedeo P., Roncaglia P., Berriman M., Hirt R.P., Mann B.J. (2005). The Genome of the Protist Parasite *Entamoeba histolytica*. Nature.

[12] Anwar T., Gourinath S. (2013). Analysis of the Protein Phosphotome of *Entamoeba histolytica* Reveals an Intricate Phosphorylation Network. PLoS ONE.

[13] Okada M., Huston C.D., Oue M., Mann B.J., Petri W.A., Kita K., Nozaki T. (2006). Kinetics and Strain Variation of Phagosome Proteins of *Entamoeba histolytica* by Proteomic Analysis. Mol. Biochem. Parasitol..

[14] LaCount M.W., Handy G., Lebioda L. (1998). Structural Origins of L(+)-Tartrate Inhibition of Human Prostatic Acid Phosphatase. J. Biol. Chem..

[15] Jakob C.G., Lewinski K., Kuciel R., Ostrowski W., Lebioda L. (2000). Crystal Structure of Human Prostatic Acid Phosphatase. Prostate.

[16] Ortlund E., LaCount M.W., Lebioda L. (2003). Crystal Structures of Human Prostatic Acid Phosphatase in Complex with a Phosphate Ion and Alpha-Benzylaminobenzylphosphonic Acid Update the Mechanistic Picture and Offer New Insights into Inhibitor Design. Biochemistry.

[17] Li J., Dong Y., Lü X., Wang L., Peng W., Zhang X.C., Rao Z. (2013). Crystal Structures and Biochemical Studies of Human Lysophosphatidic Acid Phosphatase Type 6. Protein Cell.

[18] Dhatwalia R., Singh H., Reilly T.J., Tanner J.J. (2015). Crystal Structure and Tartrate Inhibition of *Legionella pneumophila* Histidine Acid Phosphatase. Arch. Biochem. Biophys..

[19] Bechtel T.J., Weerapana E. (2017). From Structure to Redox: The Diverse Functional Roles of Disulfides and Implications in Disease. Proteomics.

[20] Sowdhamini R., Srinivasan N., Shoichet B., Santi D.V., Ramakrishnan C., Balaram P. (1989). Stereochemical Modeling of Disulfide Bridges. Criteria for Introduction into Proteins by Site-Directed Mutagenesis. Protein Eng. Des. Sel..

[21] Hudson D.A., Gannon S.A., Thorpe C. (2015). Oxidative Protein Folding: From Thiol-Disulfide Exchange Reactions to the Redox Poise of the Endoplasmic Reticulum. Free Radic. Biol. Med..

[22] Robinson P.K. (2015). Enzymes: Principles and Biotechnological Applications. Essays Biochem..

[23] Colussi F., Garcia W., Rosseto F.R., de Mello B.L.S., de Oliveira Neto M., Polikarpov I. (2012). Effect of pH and Temperature on the Global Compactness, Structure, and Activity of Cellobiohydrolase Cel7A from *Trichoderma harzianum*. Eur. Biophys. J. EBJ.

[24] Lim D., Golovan S., Forsberg C.W., Jia Z. (2000). Crystal Structures of *Escherichia coli* Phytase and Its Complex with Phytate. Nat. Struct. Biol..

[25] Golovan S., Wang G., Zhang J., Forsberg C.W. (2000). Characterization and Overproduction of the *Escherichia coli* AppA Encoded Bifunctional Enzyme That Exhibits Both Phytase and Acid Phosphatase Activities. Can. J. Microbiol..

[26] Edelheit O., Hanukoglu A., Hanukoglu I. (2009). Simple and Efficient Site-Directed Mutagenesis Using Two Single-Primer Reactions in Parallel to Generate Mutants for Protein Structure–function Studies. BMC Biotechnol..

[27] Ko K.M., Lee W., Yu J.-R., Ahnn J. (2007). PYP-1, Inorganic Pyrophosphatase, Is Required for Larval Development and Intestinal Function in *C. elegans*. FEBS Lett..

[28] Kajander T., Kellosalo J., Goldman A. (2013). Inorganic Pyrophosphatases: One Substrate, Three Mechanisms. FEBS Lett..

[29] Farquharson K.L. (2018). Life of PPi: Soluble PPases and H+-PPase Act Cooperatively to Keep Pyrophosphate Levels in Check. Plant Cell.

[30] Baykov A.A., Anashkin V.A., Salminen A., Lahti R. (2017). Inorganic Pyrophosphatases of Family II-Two Decades after Their Discovery. FEBS Lett..

[31] Lee H.S., Cho Y., Kim Y.-J., Lho T.-O., Cha S.-S., Lee J.-H., Kang S.G. (2009). A Novel Inorganic Pyrophosphatase in *Thermococcus Onnurineus* NA1. FEMS Microbiol. Lett..

[32] May A., Berger S., Hertel T., Köck M. (2011). The *Arabidopsis thaliana* Phosphate Starvation Responsive Gene AtPPsPase1 Encodes a Novel Type of Inorganic Pyrophosphatase. Biochim. Biophys. Acta BBA Gen. Subj..

[33] Huang H., Patskovsky Y., Toro R., Farelli J.D., Pandya C., Almo S.C., Allen K.N., Dunaway-Mariano D. (2011). Divergence of Structure and Function in the Haloacid Dehalogenase Enzyme Superfamily: *Bacteroides Thetaiotaomicron* BT2127 Is an Inorganic Pyrophosphatase. Biochemistry.

[34] Fahs S., Lujan P., Köhn M. (2016). Approaches to Study Phosphatases. ACS Chem. Biol..

[35] Medvedev K.E., Kinch L.N., Schaeffer R.D., Grishin N.V. (2019). Functional Analysis of Rossmann-like Domains Reveals Convergent Evolution of Topology and Reaction Pathways. PLOS Comput. Biol..

[36] Cooperman B.S., Baykov A.A., Lahti R. (1992). Evolutionary Conservation of the Active Site of Soluble Inorganic Pyrophosphatase. Trends Biochem. Sci..

[37] Heikinheimo P., Lehtonen J., Baykov A., Lahti R., Cooperman B.S., Goldman A. (1996). The Structural Basis for Pyrophosphatase Catalysis. Structure.

[38] Brock D.A., van Egmond W.N., Shamoo Y., Hatton R.D., Gomer R.H. (2006). A 60-Kilodalton Protein Component of the Counting Factor Complex Regulates Group Size in *Dictyostelium discoideum*. Eukaryot. Cell.

[39] Jang W., Gomer R.H. (2008). Combining Experiments and Modelling to Understand Size Regulation in *Dictyostelium Discoideum*. J. R. Soc. Interface.

[40] Altschul S.F., Gish W., Miller W., Myers E.W., Lipman D.J. (1990). Basic Local Alignment Search Tool. J. Mol. Biol..

[41] Marchler-Bauer A., Bo Y., Han L., He J., Lanczycki C.J., Lu S., Chitsaz F., Derbyshire M.K., Geer R.C., Gonzales N.R. (2017). CDD/SPARCLE: Functional Classification of Proteins via Subfamily Domain Architectures. Nucleic Acids Res..

[42] Blum M., Chang H.-Y., Chuguransky S., Grego T., Kandasaamy S., Mitchell A., Nuka G., Paysan-Lafosse T., Qureshi M., Raj S. (2021). The InterPro Protein Families and Domains Database: 20 Years On. Nucleic Acids Res..

[43] Webb B., Sali A. (2017). Protein Structure Modeling with MODELLER. Methods Mol. Biol. Clifton NJ.

[44] Pettersen E.F., Goddard T.D., Huang C.C., Couch G.S., Greenblatt D.M., Meng E.C., Ferrin T.E. (2004). UCSF Chimera--a Visualization System for Exploratory Research and Analysis. J. Comput. Chem..

[45] Feig M. (2016). Local Protein Structure Refinement via Molecular Dynamics Simulations with LocPREFMD. J. Chem. Inf. Model..

[46] Williams C.J., Headd J.J., Moriarty N.W., Prisant M.G., Videau L.L., Deis L.N., Verma V., Keedy D.A., Hintze B.J., Chen V.B. (2018). MolProbity: More and Better Reference Data for Improved All-Atom Structure Validation. Protein Sci. Publ. Protein Soc..

[47] Sambrook J., Russell D.W. (2001). Molecular Cloning: A Laboratory Manual.

[48] Diamond L.S., Harlow D.R., Cunnick C.C. (1978). A New Medium for the Axenic Cultivation of *Entamoeba histolytica* and Other *Entamoeba*. Trans. R. Soc. Trop. Med. Hyg..

[49] Guzman L.M., Belin D., Carson M.J., Beckwith J. (1995). Tight Regulation, Modulation, and High-Level Expression by Vectors Containing the Arabinose PBAD Promoter. J. Bacteriol..

[50] Laemmli U.K. (1970). Cleavage of Structural Proteins during the Assembly of the Head of Bacteriophage T4. Nature.

[51] Zor T., Selinger Z. (1996). Linearization of the Bradford Protein Assay Increases Its Sensitivity: Theoretical and Experimental Studies. Anal. Biochem..

[52] Baykov A.A., Evtushenko O.A., Avaeva S.M. (1988). A Malachite Green Procedure for Orthophosphate Determination and Its Use in Alkaline Phosphatase-Based Enzyme Immunoassay. Anal. Biochem..

[53] Roche D.B., McGuffin L.J. (2016). In Silico Identification and Characterization of Protein-Ligand Binding Sites. Methods Mol. Biol. Clifton NJ.

[54] McGuffin L.J., Adiyaman R., Maghrabi A.H.A., Shuid A.N., Brackenridge D.A., Nealon J.O., Philomina L.S. (2019). IntFOLD: An Integrated Web Resource for High Performance Protein Structure and Function Prediction. Nucleic Acids Res..

[55] Roche D.B., Tetchner S.J., McGuffin L.J. (2011). FunFOLD: An Improved Automated Method for the Prediction of Ligand Binding Residues Using 3D Models of Proteins. BMC Bioinform..

[56] Roche D.B., Buenavista M.T., McGuffin L.J. (2013). The FunFOLD2 Server for the Prediction of Protein-Ligand Interactions. Nucleic Acids Res..

[57] Roche D.B., Buenavista M.T., McGuffin L.J. (2012). FunFOLDQA: A Quality Assessment Tool for Protein-Ligand Binding Site Residue Predictions. PLoS ONE.

[58] Bitencourt-Ferreira G., de Azevedo W.F. (2019). Molecular Docking Simulations with ArgusLab. Methods Mol. Biol. Clifton NJ.

[59] O’Boyle N.M., Banck M., James C.A., Morley C., Vandermeersch T., Hutchison G.R. (2011). Open Babel: An Open Chemical Toolbox. J. Cheminformatics.

[60] Laskowski R.A., Swindells M.B. (2011). LigPlot+: Multiple Ligand-Protein Interaction Diagrams for Drug Discovery. J. Chem. Inf. Model..

[61] Salentin S., Schreiber S., Haupt V.J., Adasme M.F., Schroeder M. (2015). PLIP: Fully Automated Protein–Ligand Interaction Profiler. Nucleic Acids Res..

