# Supplementary material for: Structure–Function Relationship Study of a Secretory Amoebic Phosphatase: A Computational-Experimental Approach
Source: Int J Mol Sci. 2021 Feb 22;22(4):2164. doi: 10.3390/ijms22042164 (PMC7926622; doi:10.3390/ijms22042164)
Supplement: Supplementary file 1 [file ijms-22-02164-s001.pdf]

International Journal of Molecular Sciences

Supplementary Material

Structure-Function Relationship Study of an Amoebic Secretory Phosphatase: A Computational-Experimental Approach

Celina Terán-Ramírez, Rosa E. Mares-Alejandre*, Ana L. Estrada-González, Patricia L. A. Muñoz-Muñoz and Marco A. Ramos-Ibarra*

Biotechnology and Biosciences Research Group, Faculty of Chemical Sciences and Engineering, Autonomous University of Baja California, Tijuana, BCN, Mexico.

* Correspondence: rmares@uabc.edu.mx (R.E.M.A.); mramos@uabc.edu.mx (M.A.R.I.).

```
EhHAPp49    MLWLLLLFQLSLGDLTYCEVPEFTFKPVDGYKPILLQMVFRHGDRSPWLTYKGDQATYNCDISQQLRFLSAQGMESYEFHHIKTEVDKEK
HuPAP_1ND5  -----------------------------------VTLVFRHGDRSPIDTFPTDPIKE--------------------------------
HuLAP_4JOB  ----------------------------------MVQVVFRHGARSPLKPLPLEEQV---EWNPQLLEVPPQTQFDYTVTNLAGGPKPYS
LpHAP_5CDH  ------------------------------------------------------------------------------------------

EhHAPp49    M--VFAKDNMYGGSCQMGQLTRKGLNQLATLGDKVRANYVGDNNFLPTYMNISDIYVRSTKVWRVIQSAESFLQHLYPSYSRDPD-----
HuPAP_1ND5  ----------SSWPQGFGQLTQLGMEQHYELGEYIRKRYRK---FLNESYKHEQVYIRSTDVDRTLMSAMTNLAALFPPEGVSIW-----
HuLAP_4JOB  PYDSQYHETTLKGGMFAGQLTKVGMQQMFALGERLRKNYVEDIPFLSPTFNPQEVFIRSTNIFRNLESTRCLLAGLFQCQKEGPI-----
LpHAP_5CDH  ----------------LGQLTAEGMQQEYKMGVAFRKKYIEELHLLPEHYEYGTIYVRSTDYARTLMSAQSLLMGLYPPGTGPSIPAGTS

EhHAPp49    ------ARIRINTVPSEIEYAVYNDHGMCPREAELEKELFDKWFVEELNLRHKEPYASILAKAERFFGVVNYNWYDSYFDILQEVQCNKL
HuPAP_1ND5  NPILLWQPIPVHTVPLSEDQLLYLPFRNCPRFQELESETLKSEEFQKR----LHPYKDFIATLGKLSGLHGQDLFGIWSKVYDPLYCESV
HuLAP_4JOB  ---------IIHTDE-ADSEVLYPNYQSCWSLRQRTRGRRQTASLQPG-------ISEDLKKVKDRMGIDSSDK-VDFFILLDNVAAEQA
LpHAP_5CDH  ALPHAFQPIPVFSAPSKYDEVIIQQVDRKERKKLMEQYVFSTREWQQK----NNELKDKYPLWSRLTGINIDTL-EDLETVGHTLYVHQI

EhHAPp49    EWPCIDTESGEKECFTEEEFNKLVELVYHDGVYRFFDNDTIPLARLDAG----WFLRDILSFQQMKYEGKTNVKYTHFHAHDTTIYPLVS
HuPAP_1ND5  HNFTL------PSWATEDTMTKLRELSELSLLSLYGIHKQKEKSRLQGG----VLVNEILNHMKRATQIPSYKKLIMYSAHDTTVSGLQM
HuLAP_4JOB  HNL--------PSCPMLKRFARMIEQRAVDTSLYILPKEDRESLQMAVGPFLHILESNLLKAMDSATAPDKIRKLYLYAAHDVTFIPLLM
LpHAP_5CDH  HNAPM------PEGLASNDIETIINSAEWAFM---AQEKPQQIANVYSS----KLMTNIADYLNSGSMKKSKLKYVLLSAHDTTIASVLS

EhHAPp49    LLEGDYSSWPPYASYIIFEMYEK--ESEYYIRVGYNNKLL------DLNFCSKDENGMCKWKSFFDHMSKKVP--KSSDCNAKNQ
HuPAP_1ND5  ALDVYNGLLPPYASCHLTELYFE--KGEYFVEMYYRNETQHEPYPLMLPGCSP----SCPLERFAELVGPVIPQDWSTECMTTN-
HuLAP_4JOB  TLGIFDHKWPPFAVDLTMELYQHLESKEWFVQLYYHGKEQ------VPRGCPD---GLCPLDMFLNAMS----------------
LpHAP_5CDH  FLGAPLEKSPPYASNVNFSLYDN--GANY-----YTVKITYNGNPVLIPACGG---SVCELQQLVNLVHDSKNL-----------
```

Figure S1. Multiple sequence alignment of the protein sequences of *Eh*HAPp49 and three structural homologs: HuPAP, human prostatic acid phosphatase (PDB Entry 1ND5); HuLAP, human lysophosphatidic acid phosphatase type 6 (PDB Entry 4JOB); and LpHAP, *Legionella pneumophila* HAP (PDB Entry 5CDH). The alignment was performed using Chimera's Multalign Viewer tool. Similar residues are shaded gray, while identical residues are boxed.

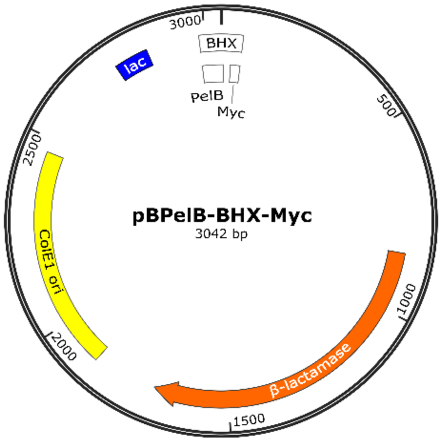

Figure S2. Schematic depiction of pBPelB-BHX-Myc, a pBluescript-based plasmid allowing gene fusion of a periplasmic targeting sequence (PelB) and a molecular tag peptide (Myc) to the N- and C-termini ends of recombinant proteins. BHX represents a mini MCS, which includes the BamHI, HindIII, and XhoI restriction sites. While ColE1 ori (yellow) functions as the autonomous replication sequence, encoded β-lactamase (orange) as the selection marker (Amp^R), and lac promoter (blue) as the regulatory region.

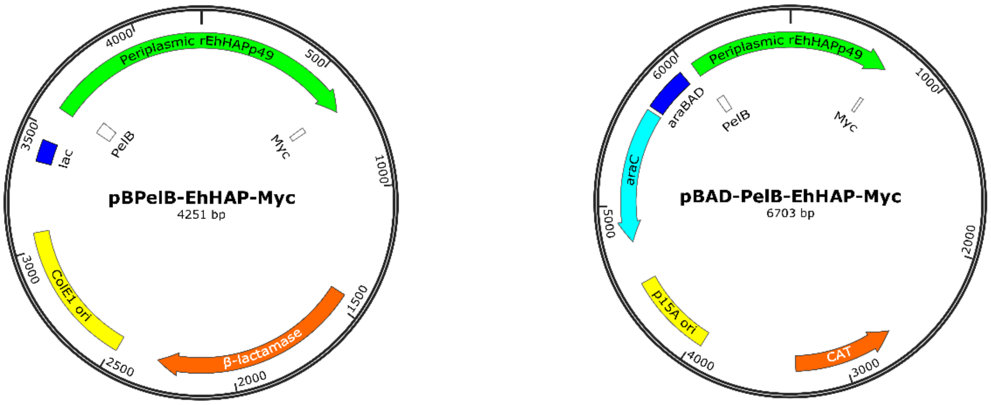

(**a**) (**b**)

Figure S3. (**a**) Schematic depiction of pBPelB-EhHAP-Myc, a pBluescript-based plasmid encoding the *Eh*HAPp49 as a Myc-tagged periplasmic protein under the control of lac promoter (blue). ColE1 ori (yellow) and β-lactamase (orange) function as described for the parental plasmid, pBPelB-BHX-Myc (Supp. Fig. 1). (**b**) Schematic depiction of pBAD-PelB-EhHAP-Myc, a pBAD33-based plasmid encoding the *Eh*HAPp49 as a Myc-tagged periplasmic protein under the control of araBAD promoter (blue). While p15A ori (yellow) functions as the autonomous replication sequence, encoded chloramphenicol acetyl-transferase, CAT (orange), as the selection marker (Cm^R), and AraC (turquoise) as the regulatory protein.

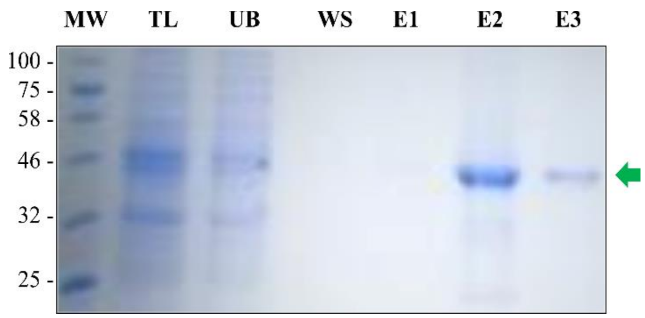

Figure S4. Analysis of IMAC fractions by 13.5% SDS-PAGE. Lanes: MW, molecular weight markers (kDa; on the left side); TL, total lysate; UB, unbound; WS, wash; E1-E3, elutions. Gel stained with Coomassie Brilliant Blue. On the right side, a green arrow indicates the relative mobility of r*Eh*HAPp49.
